# Intracerebral Hemorrhage in a Middle-Aged Male With Classical Ehlers-Danlos Syndrome in the Absence of Macrovascular Lesions: A Case Report

**DOI:** 10.7759/cureus.110138

**Published:** 2026-06-02

**Authors:** Saud A Daghreeri, Abdullah A Laftal, Ghaida Al Hunaif, Afnan K Sibyani

**Affiliations:** 1 Neurology, Aseer Central Hospital, Abha, SAU; 2 Neuroradiology, Aseer Central Hospital, Abha, SAU; 3 College of Medicine, King Khalid University, Abha, SAU; 4 Neurology, King Abdulaziz Medical City, Jeddah, SAU

**Keywords:** cerebrovascular complications, ehlers-danlos syndrome, intracerebral hemorrhage, vascular fragility, venous leakage

## Abstract

Ehlers-Danlos syndrome (EDS) is a heterogeneous connective tissue disorder characterized by tissue fragility, joint hypermobility, and variable vascular involvement. Although intracerebral hemorrhage (ICH) is classically associated with vascular EDS, emerging evidence suggests that cerebrovascular complications may occur across multiple EDS subtypes, even in the absence of overt vascular malformations. We report a 39-year-old Saudi male with genetically confirmed classical Ehlers-Danlos syndrome (cEDS) associated with COL5A1 variants who presented with acute decreased level of consciousness, expressive aphasia, and mild right-sided weakness. Brain computed tomography demonstrated a left hemispheric ICH. Given the patient’s young age and underlying connective tissue disorder, extensive vascular evaluation was performed. Digital subtraction angiography showed no evidence of aneurysm, arteriovenous malformation, dural fistula, or cerebral venous thrombosis. Subsequent magnetic resonance imaging revealed pial meningeal enhancement and engorged cortical vessels adjacent to the hemorrhage, findings suggestive of vascular changes likely related to underlying vascular fragility, with no direct evidence of venous leakage. The patient was managed conservatively with multidisciplinary care and rehabilitation, resulting in gradual neurological improvement and radiological stability. This case highlights that spontaneous ICH can occur in patients with EDS even in the absence of identifiable macrovascular lesions and underscores the potential role of underlying microvascular fragility in the pathogenesis of hemorrhagic complications. Increased awareness of cerebrovascular involvement across the EDS spectrum is essential for early recognition and appropriate management.

## Introduction

Ehlers-Danlos syndrome (EDS) is a group of genetically diverse connective tissue disorders, marked by skin hyperextensibility, joint hypermobility, and vascular fragility [1]. While vascular Ehlers-Danlos syndrome (vEDS), caused by COL3A1 mutations, is primarily associated with arterial rupture and aneurysms [2], recent studies have shown that non-vascular EDS subtypes also carry significant cerebrovascular risk, including intracerebral hemorrhage (ICH), arterial dissections, and aneurysms [3]. This challenges the traditional view that only vEDS poses a substantial cerebrovascular risk, highlighting that all EDS subtypes can demonstrate varying degrees of vascular vulnerability.

Hemorrhagic complications in non-vascular EDS subtypes are thought to result from microvascular fragility and connective tissue-related vascular vulnerability.

Although neurovascular manifestations in EDS are rare, they are increasingly recognized and may include intracerebral, subarachnoid, and epidural hemorrhages [4]. Additionally, hemostatic abnormalities such as easy bruising and coagulation or platelet dysfunction may further contribute to bleeding risk [5,6]. In pediatric cases, even pulmonary hemorrhage has been reported in non-vascular EDS subtypes [7]. The inherent vascular fragility in EDS also increases the risk of complications during invasive procedures such as surgery and endovascular interventions [3,8].

We report a case of a 39-year-old male with classical Ehlers-Danlos syndrome (cEDS) associated with COL5A1 variants, contributing to connective tissue fragility, who presented with spontaneous ICH in the absence of identifiable vascular malformations on angiographic evaluation.

## Case presentation

A 39-year-old Saudi male with a known diagnosis of EDS, specifically genetically confirmed cEDS associated with COL5A1 variants diagnosed during childhood at King Faisal Specialist Hospital and Research Centre (KFSHRC) following evaluation for generalized joint laxity, initially presented to a peripheral community hospital with acute onset of decreased level of consciousness and inability to speak.

According to family members, the symptoms developed suddenly while the patient was at rest. There was no preceding trauma, seizure activity, headache, fever, constitutional symptoms, recent infection, anticoagulant use, or illicit drug exposure. His medical history was otherwise unremarkable, with no prior cerebrovascular events.

On initial examination, the patient was somnolent but arousable to painful stimuli. Neurological examination revealed marked expressive aphasia with preserved comprehension of simple commands, in addition to mild right-sided hemiparesis involving both upper and lower extremities. Pupils were equal and reactive to light, and no clear cranial nerve deficits were identified. Vital signs were within normal limits, without evidence of hypertension, fever, or hypoxia. Cardiovascular and respiratory examinations were unremarkable, and initial laboratory investigations showed no major abnormalities.

Initial laboratory investigations revealed a platelet count of 127 (150-400 ×10^9/L), international normalized ratio (INR) of 1.1 (0.9-1.2), prothrombin time (PT) of 14 seconds (11-13.5 seconds), and partial thromboplastin time (PTT) of 31 seconds (25-35 seconds).

Urgent non-contrast computed tomography (CT) of the brain demonstrated a left hemispheric ICH without significant midline shift or hydrocephalus. Given the patient’s relatively young age and underlying connective tissue disorder, the hemorrhage was considered atypical and suspicious for an underlying vascular etiology. Accordingly, the patient was transferred to our tertiary care center for further neurological, vascular, and neurosurgical evaluation.

Upon arrival, repeat neuroimaging demonstrated an intraparenchymal hemorrhage involving the left parieto-occipital region with mild mass effect and minimal intraventricular extension, without significant midline shift or acute hydrocephalus. CT angiography (CTA) did not demonstrate evidence of arteriovenous malformation (AVM), arteriovenous fistula (AVF), or other major vascular abnormalities (Figure 1).

**Figure 1:**
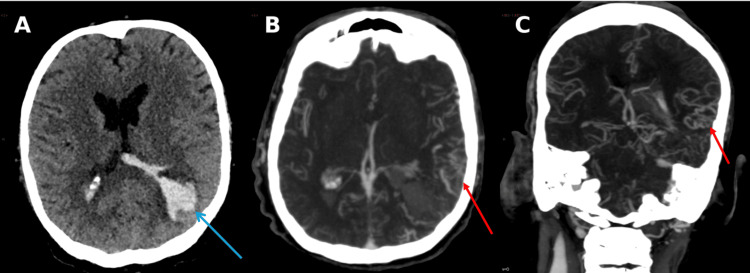
Brain CT scan. Unenhanced CT scan of the brain (A) showing intraparenchymal hemorrhage involving the left occipito-inferior parietal region, exerting a mild mass effect and extending into the ventricular system, with no significant midline shift or acute hydrocephalus (blue arrow). CT angiography of the brain (B and C) demonstrates the hemorrhage without evidence of an arteriovenous malformation or arteriovenous fistula. There is no overt contrast enhancement to suggest an underlying mass, and the deep veins remain patent. Notably, prominent cortical vessels are observed, appearing more pronounced compared to the contralateral hemisphere (red arrows), a finding that is nonspecific and may reflect regional hemodynamic alteration in the setting of acute hemorrhage.

Given the patient’s stable neurological status and the absence of significant mass effect, conservative management was favored over surgical intervention.

The patient remained under close neurosurgical observation with serial neurological assessments demonstrating no evidence of clinical deterioration. Laboratory investigations during the first four days of hospitalization showed a gradual decline in red blood cell count (5.08 to 4.45 ×10^6/μL) and hematocrit (46.26% to 39.53%), interpreted as a mild dilutional or post-illness effect in the absence of ongoing bleeding. Mild leukocytosis present on admission resolved spontaneously, consistent with a physiological stress response. Electrolyte levels, including serum sodium, remained within normal limits throughout hospitalization.

Following clinical and radiological stabilization, the patient was transferred to the neurology service for continued conservative management. A multidisciplinary care approach involving neurology, rehabilitation medicine, intensive care, and internal medicine teams was implemented, with emphasis on neurological recovery, prevention of complications, and early mobilization.

Given the absence of an identifiable etiology for the hemorrhage, further neurovascular evaluation was pursued. Brain magnetic resonance imaging (MRI) demonstrated a subcortical left parieto-occipital hemorrhage with minimal intraventricular extension and patent major intracranial vessels without evidence of vascular malformation (Figure 2).

**Figure 2:**
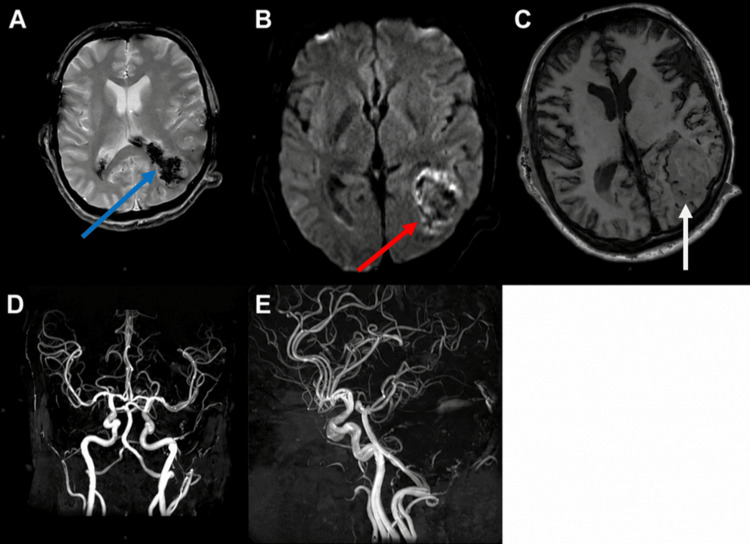
Non-contrast brain MRI. Susceptibility-weighted imaging (A) demonstrates the hemorrhage extending from the subcortical region into the ventricular system (blue arrow). Diffusion-weighted imaging (B) reveals peripheral restricted diffusion (red arrow). T1-weighted imaging (C) shows an isointense hematoma with a bright linear signal indicative of early peripheral T1 shortening, alongside a medial signal void representing a prominent cortical vessel (white arrow). Time-of-flight magnetic resonance angiography (D and E) confirms patency of the major intracranial vessels without aneurysmal dilatation, arteriovenous malformation, or dural fistula.

Subsequent digital subtraction angiography (DSA) demonstrated normal intracranial vascular arborization without evidence of aneurysm, AVM, dural arteriovenous fistula, or other structural vascular abnormalities (Figure 3).

**Figure 3:**
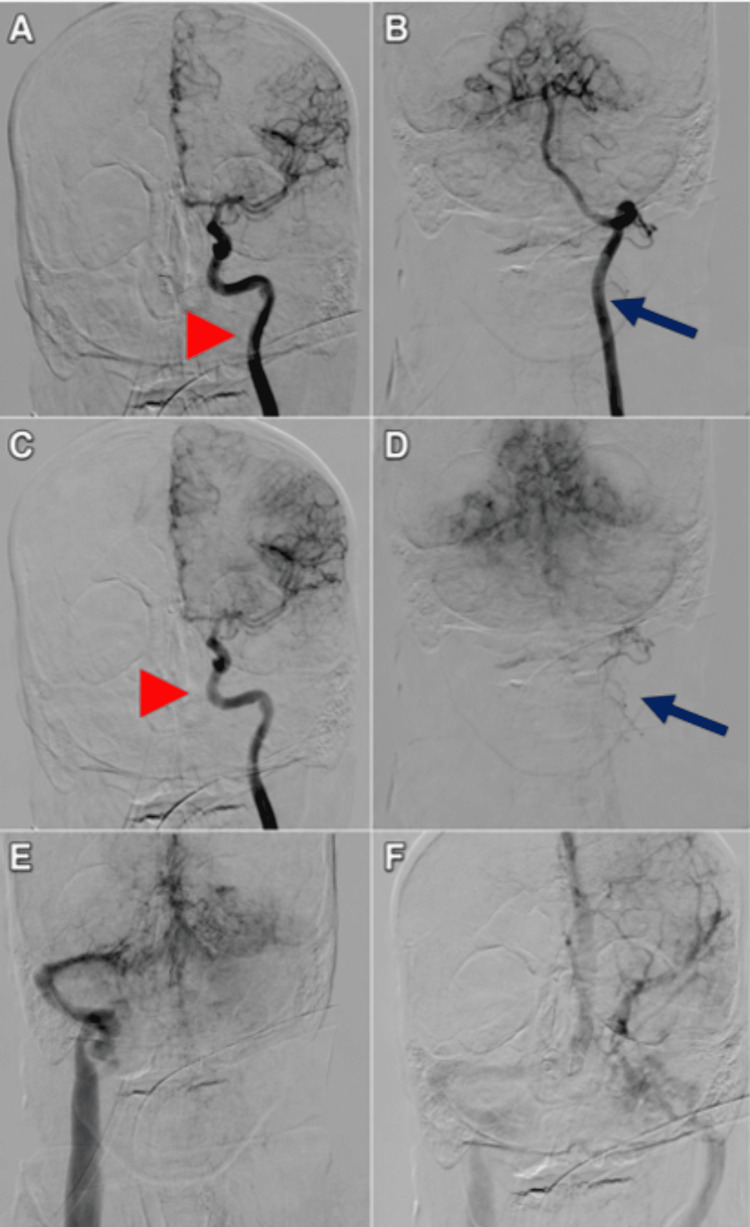
Digital subtraction angiography (DSA). Composite anteroposterior (AP) views of the left internal carotid artery (A and C) (red arrowheads), vertebral artery (B and D) (blue arrows), and jugular vein angiography (E and F). The images encompass the arterial, capillary, and venous phases. The studies demonstrate normal intracranial vascular arborization, with no angiographic evidence of an underlying aneurysm, arteriovenous malformation, dural arteriovenous fistula, or tumor blush to account for the left parieto-occipital hemorrhage.

Further evaluation with contrast-enhanced MRI and magnetic resonance venography (MRV) demonstrated pial meningeal enhancement and engorged cortical vessels surrounding the hemorrhagic region, without evidence of venous sinus thrombosis or underlying neoplastic lesions. These findings were nonspecific and likely represent reactive vascular changes in the setting of acute ICH, without evidence of venous obstruction or structural lesion (Figure 4).

**Figure 4:**
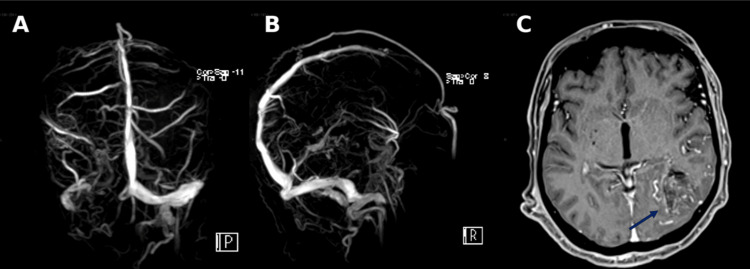
Follow-up brain MRI performed to confirm patency of the dural sinuses and exclude an underlying mass. Phase-contrast magnetic resonance venography (A and B) demonstrates patent dural sinuses with no evidence of sinus thrombosis. Post-contrast MRI (C) confirms the absence of a suspicious enhancing underlying mass (blue arrow). Subtle pial meningeal enhancement and engorged cortical vessels are noted adjacent to the hemorrhagic region, findings that are nonspecific and may reflect reactive vascular changes in the setting of acute intracerebral hemorrhage, rather than a primary vascular etiology, without evidence of venous obstruction or structural lesion.

Following neurological stabilization, the patient was transferred to a private rehabilitation facility closer to his family, where he underwent an intensive eight-day rehabilitation program. Gradual improvement in expressive language function and right-sided motor strength was observed. He remained medically stable throughout rehabilitation, with no recurrent neurological events or additional complications. Follow-up neuroimaging demonstrated stable evolution and partial resolution of the ICH without interval development of new abnormalities. The patient was subsequently discharged home in stable condition with structured outpatient follow-up through neurology and rehabilitation services.

The patient’s history of EDS had been established several years earlier through genetic testing at KFSHRC, performed due to chronic lower-limb joint laxity. Despite this diagnosis, he had no prior vascular complications. This case highlights a spontaneous ICH in a patient with EDS in the absence of demonstrable vascular malformation, suggesting an underlying predisposition to microvascular fragility inherent to connective tissue disorders.

## Discussion

Spontaneous ICH in patients with EDS represents a rare but clinically significant manifestation of the disease spectrum. Although vascular complications have traditionally been associated with vEDS, emerging evidence suggests that cerebrovascular events may also occur across other EDS subtypes, reflecting a broader underlying vascular fragility than previously recognized. This evolving understanding challenges the classical subtype-specific risk stratification and highlights the need to consider EDS as a systemic connective tissue disorder with potential neurological and vascular implications in all its forms.

This case presents a 39-year-old male with genetically confirmed cEDS associated with COL5A1 variants who developed spontaneous ICH in the absence of vascular malformations or venous thrombosis. It highlights the growing recognition that vascular fragility is not limited to vEDS but extends across all EDS subtypes, with studies documenting ICH in conditions such as classical, kyphoscoliotic, and musculocontractural EDS [3]. This suggests that microvascular vulnerability may be a common pathological feature in EDS, regardless of subtype.

The pathophysiology of vascular fragility in EDS is linked to defects in collagen and extracellular matrix proteins, such as COL3A1 and COL5A1, which compromise blood vessel integrity [2]. This increases the risk of spontaneous rupture even in the absence of overt vascular malformations [5,3]. In subtypes such as classical and hypermobile EDS, where major vascular events are less common, connective tissue defects often manifest as easy bruising or delayed wound healing [8]. In this case, the hemorrhage likely resulted from underlying microvascular fragility, potentially triggered by subtle physiological stressors, despite the absence of hypertension or coagulopathy.

While DSA remains the gold standard for detecting aneurysms or AVMs, it carries significant procedural risks in EDS patients due to inherent vascular fragility [7]. In this patient, the absence of macrovascular lesions on angiography suggests bleeding at the microvascular or capillary level, which is consistent with reported cases of ICH in non-vascular EDS patients without identifiable vascular abnormalities [4].

Management of hemorrhagic complications in EDS requires a multidisciplinary approach, particularly given the risks associated with invasive interventions [8]. In this case, conservative management, including close neurological monitoring and supportive care, was appropriate. Early rehabilitation, including physical, occupational, and speech therapy, played an important role in functional recovery [8]. Similar reports have highlighted the benefit of early rehabilitation in improving outcomes following ICH in patients with connective tissue disorders.

Long-term care in EDS should focus on reducing the risk of recurrent vascular events through regular surveillance and risk factor modification. Non-invasive imaging modalities such as MRI or magnetic resonance angiography may be considered for patients with prior hemorrhagic events or confirmed EDS. Additionally, strict control of modifiable factors such as blood pressure remains essential in minimizing future vascular complications [9,10]. Notably, the patient had no prior history of vascular complications despite longstanding genetically confirmed EDS, emphasizing that serious cerebrovascular events may occur even in clinically stable patients without previous vascular manifestations.

Overall, this case reinforces that spontaneous ICH can occur in EDS beyond the vascular subtype, even in the absence of overt vascular lesions, highlighting the potential role of underlying microvascular fragility rather than a specific venous mechanism in the pathogenesis of hemorrhagic complications.

## Conclusions

This case reinforces that spontaneous ICH can occur in EDS beyond the vascular subtype, even in the absence of overt vascular lesions, highlighting underlying microvascular fragility. Clinicians should consider EDS in the differential diagnosis of spontaneous ICH, especially in younger patients, and adopt multidisciplinary care, conservative management, careful procedural planning, and attention to modifiable risk factors to optimize outcomes.

Importantly, the proposed mechanism of “venous leakage” remains speculative and is based on exclusion of identifiable macrovascular pathology rather than direct imaging confirmation, and should therefore be interpreted cautiously. Further research is needed to guide cerebrovascular risk stratification and management strategies across all EDS subtypes.
